# Mechanical Performance of Nonabsorbable Monofilament Suture Materials Tied with Different Suturing Techniques Under Various Knot Configurations: An In Vitro Study

**DOI:** 10.3390/jfb16120428

**Published:** 2025-11-22

**Authors:** Nuri Mert Taysi, Aysegul Erten Taysi, Pinar Ercal, Soner Sismanoglu

**Affiliations:** 1 Department of Oral and Maxillofacial Surgery, Faculty of Dentistry, Istanbul University-Cerrahpaşa, 34098 Istanbul, Turkey; nurimert.taysi@iuc.edu.tr; 2 Department of Oral and Maxillofacial Surgery, Faculty of Dentistry, Istanbul Aydın University, 34295 Istanbul, Turkey; 3 Department of Oral and Maxillofacial Surgery, Faculty of Dentistry, Istanbul Health and Technology University, 34445 Istanbul, Turkey; pinar.ercal@istun.edu.tr; 4 Department of Restorative Dentistry, Faculty of Dentistry, Istanbul University-Cerrahpaşa, 34098 Istanbul, Turkey; soner.sismanoglu@iuc.edu.tr

**Keywords:** artificial saliva, monofilament suture materials, material testing, nonabsorbable suture materials, sutures, suture techniques, wound closure techniques

## Abstract

This study compared widely used nonabsorbable and monofilament suture materials tied with three different configurations and two different suture techniques. Three suture materials (polytetrafluoroethylene, polypropylene, and nylon) were tied with either Laurell–Gottlow or the horizontal mattress suturing techniques using three different knot configurations: A (2 = 1 = 1 = 1 = 1), B (2 × 1 = 1 = 1 = 1), and C (1 × 2 = 1 = 1 = 1) on an experimental platform manufactured using a three-dimensional printer. Specimens underwent microtensile testing to determine maximum load failure and elongation rates at baseline and after 7 days of artificial saliva immersion. The Laurell–Gottlow yielded significantly lower elongation rates and higher failure load than the horizontal mattress suturing technique using nylon and polypropylene sutures at both time points (*p* < 0.001). Nylon had a significantly higher failure load and elongation than polypropylene and polytetrafluoroethylene at baseline for both suturing techniques and all three knot configurations (*p* < 0.001). Configuration C had low failure load values following immersion for all suture materials when using horizontal mattress suturing. Configuration A demonstrated superior failure load following the immersion period for all materials using both techniques. The polytetrafluoroethylene suture remained more stable over time. These findings indicate that the Laurell–Gottlow suturing technique with Configuration A provides better mechanical resistance to external forces when using nonabsorbable monofilament suture materials.

## 1. Introduction

Proper wound healing after a surgical intervention is dependent on immobilization of the wound area, which necessitates an appropriate suture material tied with a suitable technique. With an optimal suturing method, primary healing can be achieved in cases of critical importance, such as tissue regeneration, implant dentistry, or microsurgical operations for improving soft tissue esthetics [1].

The selection of suturing techniques varies in oral surgery as each technique offers specific advantages and disadvantages. Understanding the wound anatomy and the properties of a selected technique is essential for achieving success in tissue approximation. The most common suturing technique in dentistry is the simple-loop interrupted suturing technique, which is applied to close flaps and incisions [2]. However, when this technique is used in closing elevated papilla after flap operations, craters may form at the treated sites [1]. To overcome such challenges and achieve interdental soft tissue approximation, the Laurell–Gottlow suturing technique may be used, which entails a horizontal mattress suture in combination with a simple interrupted suture [3,4]. This technique relieves mechanical tension directed to the interdental tissue, allows precise papilla placement, and enables secure flap closure. It is successfully used in a variety of regenerative procedures, including the sausage technique and alveolar ridge preservation [5,6,7].

Beyond the suturing technique itself, several fundamental factors, such as suture material’s properties, knot configuration, suture size, and number of throws, critically influence suturing success in terms of knot security and mechanical behavior [8]. Sutures are classified according to multiple criteria [9]. The primary distinction is based on being absorbable or nonabsorbable [10]. Additional distinctions include natural versus synthetic origin, monofilament versus multifilament, coated versus uncoated, and dyed versus undyed [9,10]. Beyond conventional suture categories, stem cell-seeded sutures and smart sutures are emerging technologies that merit acknowledgment; however, these advanced materials are not yet in clinical usage [9,11,12].

Monofilament and nonabsorbable sutures are advantageous, as they are less likely to cause tissue reactions and harbor smaller microbial loads, with nylon sutures showing comparatively less aggregation of bacteria [13]. Beyond mechanical considerations, antimicrobial performance represents a critical factor in suture selection for oral cavity repairs. Pcheliakov et al. demonstrated in their clinical study that bacterial colonization patterns differ markedly between suture structures, with monofilament materials showing significantly lower bacterial adherence (*p* < 0.05) [14]. However, practical challenges exist due to the greater memory of monofilament sutures, which may result in the unraveling of the knot if not tied correctly.

Knot configuration is another critical aspect of suturing, as it is essential to the stability of the suture. Various studies have investigated the number of throws and knot configurations using different types of suture materials [15,16,17]. A report on the knot failure load of different suture materials indicated that failure depended on both the material and the knot configuration used, whereas the physical conditions showed no effect [18]. However, to the best of our knowledge, there is no comparative study in the literature that evaluated the effects of different knot configurations used in the Laurell–Gottlow and horizontal mattress techniques with monofilament suture materials.

There is a lack of detailed evaluations of how specific knot configurations affect the mechanical performance of different suturing techniques [15,16,17,18]. This knowledge gap has resulted in inconsistent clinical practices, with technique and configuration selection often relying on surgeon preferences rather than accurate mechanical performance data. The aim of the present study was to evaluate different knot configurations in combination with two different suturing techniques used in periodontal surgery for nonabsorbable monofilament sutures at different time points under the same in vitro conditions.

## 2. Materials and Methods

### 2.1. Study Design and Sample

In this in vitro experimental study, the properties of three different nonabsorbable and monofilament suture materials (Table 1) of USP 4-0 caliber were evaluated in three-knot configurations using the Laurell-Gottlow or the horizontal mattress suturing technique under immersion in artificial saliva.

**Figure 1:**
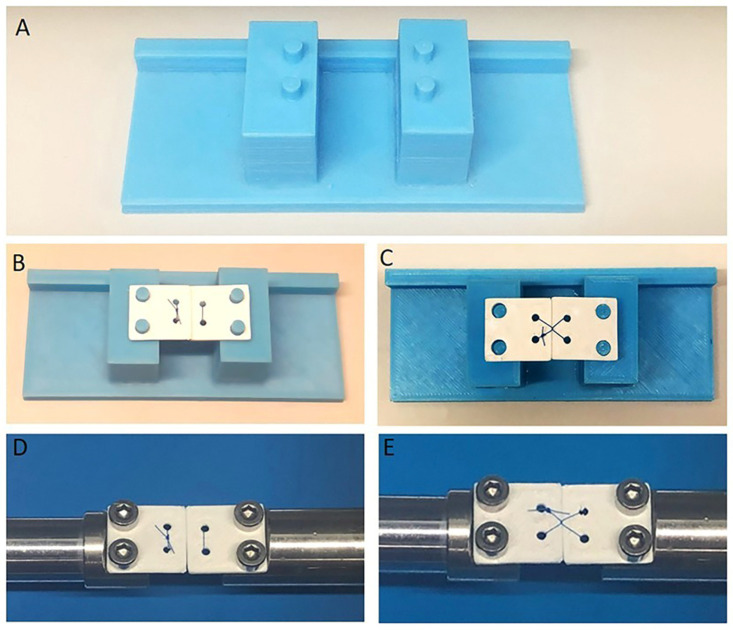
Preparation for the mechanical testing. (**A**) Experimental platform based on the testing bed of a microtensile testing device. (**B**) Plates (18 × 18 mm) serving as jaws were used to demonstrate the horizontal mattress suturing technique on the experimental platform. (**C**) Application of the Laurell–Gottlow suturing technique on plates; (**D**,**E**) Plates installed on the gripper holder of the testing bed.

An experimental platform (Figure 1A) was designed, comprising the testing bed of a microtensile testing device to which two square plates were attached as jaws to simulate a suture area. The plates (18 × 18 mm) were produced using a three-dimensional printer (Creality Ender-3 Pro, Shenzhen Creality 3D Technology Co., Ltd., Shenzhen, China) and acrylonitrile butadiene styrene filament and were designed with four holes. Two of the holes, intended for screws, were used to secure the jaws to the testing bed, and the other two holes, which were placed 4 mm apart and 3 mm from the outer edges, were utilized as suture eyelets. All specimens and fixtures were stored and tested in controlled laboratory conditions within the standard ranges for the mechanical testing of dental materials (ISO 291: Atmosphere 23 (23 °C room temperature, 50% relative humidity)).

The sample size calculation was based on a review of comparable studies, selecting the approach yielding the largest sample size according to the planned statistical methods. G*Power 3.1.9.2 [19] with α = 0.05 (95% confidence level) and a standardized effect size of 2.2634 derived from a similar study [20]; where 11.9 N with a standard deviation of 3.5 for PTFE suture under mattress technique was used. Using a target power of 0.95, the minimum required sample size was determined to be 7 per group. Considering the possibility of technical errors during the experimental procedure, the sample size per group was increased from 7 to 10.

In total, one experimental platform and 720 individual square plates were 3D-printed. The plates were assembled into 360 pairs, with each pair mounting one tied suture-knot specimen. This yielded 36 experimental groups (3 materials × 2 techniques × 3 configurations × 2 time points), with 10 specimens per group for a total of 360 specimens. For each suture material, 120 specimens were prepared: 60 using the Laurell–Gottlow technique and 60 using the simple interrupted technique. Within each material–technique combination (*n* = 60), the specimens were further subdivided by knot configuration (n = 20 per configuration: A, B, and C) and aging time point (*n* = 10 for baseline, *n* = 10 for 7 days). The specimens were randomly assigned to the experimental groups to minimize systematic biases.

**Figure 2:**
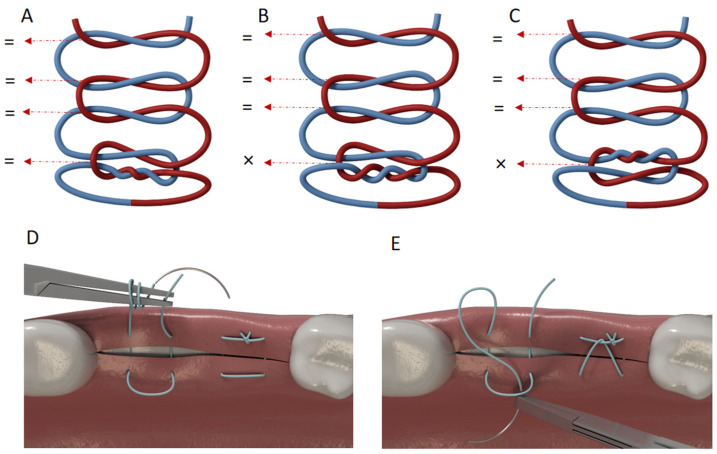
Illustrations of the studied knot configurations and suturing techniques. (**A**) 2 = 1 = 1 = 1= 1 (Configuration A), (**B**) 2 × 1 =1 = 1 = 1 (Configuration B), (**C**) 1 × 2 = 1 = 1 = 1 (for Configuration C). “=” indicates a throw in the opposite direction to the previous throw, whereas “×” indicates a throw in the same direction. (**D**) The horizontal mattress and (**E**) Laurell–Gottlow suturing techniques. All illustrations were created by A.E.T. using 3dsmax (Autodesk, Inc., San Francisco, CA, USA).

Each set of jaws was tied together on the platform (Figure 1B–E) to evaluate the properties of the selected suture materials in three-knot configurations using the Laurell–Gottlow or the horizontal mattress suturing technique. All knots were tied by one researcher (AET) using the designed platform to ensure standardization. After the suture strands were tied (Figure 2A–E), the tail lengths were cut to 3 mm long, as recommended by previous studies [15,21,22,23]. While the knots were being tied using a Mathieu needle holder, tension was maintained on the suture material manually with the aid of a custom-made platform by one experienced operator (AET, who has 14 years of surgical experience) but was not formally measured, which reflects standard clinical practice as it has been shown that instrumental tension measurement does not significantly affect knot security [24]. The artificial saliva solution was prepared at the Altinbas University Faculty of Pharmacy according to a previously published formula [25,26]: 0.7 mmol/L CaCl_2_, 0.2 mmol/L MgCl_2_·6H_2_O, 4.0 mmol/L KH_2_PO_4_, 30 mmol/L KCl, 0.3 mmol/L NaN_3_, and 20 mmol/L HEPES buffer were sequentially dissolved in deionized water. The pH was adjusted to 7.0–7.4, and the solution was filter-sterilized (0.22 μm). The pH of the artificial saliva was then adjusted to 6.7 [25,26]. No agitation or replacement of the solution was performed during the 7-day immersion period. A pair of plates for each suture material and technique was immediately tested to failure on day 0, and the other half of the plates was immersed in artificial saliva at 37 °C for 7 days before evaluation.

### 2.2. Study Variables

The predictor variables were the suture material, suturing technique, knot configuration, and immersion period. For the first variable, three different suture materials (Table 1) were tested: polytetrafluoroethylene (PTFE; Cytoplast, Osteogenics Biomaterials, Lubbock, TX, USA), polypropylene (Propilen, Dogsan, Trabzon, Turkey), and nylon (Resolon, Resorba Medical GmbH, Nürnberg, Germany). For the second variable, two suturing techniques were applied: the Laurell–Gottlow suturing technique and the horizontal mattress suturing technique. The third variable, knot configuration, included three tying techniques: a surgeon’s knot plus three square knots (2 = 1 = 1 = 1 = 1), a surgeon’s knot granny plus three square knots (2 × 1 = 1 = 1 = 1), and a reverse surgeon’s knot granny plus three square knots (1 × 2 = 1 = 1 = 1), hereafter referred to as Configurations A, B, and C, respectively (Figure 2A–C). The surgeon’s knot granny and reverse surgeon’s knot granny were selected because they are recommended by the manufacturers of the PTFE and nylon suture materials, respectively. Three square knots were additionally tied to maintain proper knot security as advised by Muffly et al. [13]. The final variable was the immersion period. A duration of 7 days was chosen for immersion because this amount of time is a common preference among clinicians for the removal of stitches or postoperative follow-up visits.

### 2.3. Data Collection Methods

This in vitro study was conducted in the Mechanical Testing Laboratory of Altinbas University in Istanbul, Turkey. The mechanical behavior of suture materials tied with the Laurell–Gottlow or the horizontal mattress suturing technique using different knot configurations was evaluated using a micro-tensile testing device (MOD Dental, Esetron Smart Robotechnologies, Ankara, Turkey) at baseline and on day 7. For the failure analysis, each pair of square plates was fixed to the testing device with four Allen screws and then stretched with gradually increasing force at a crosshead speed of 5 mm/min. No preload was applied prior to testing. Displacement was measured grip-to-grip via crosshead movement using a 500 N load cell. Maximum load to failure and elongation at failure were recorded in N and µm, respectively.

### 2.4. Statistical Analysis

Failure load and elongation outcomes were analyzed using a unified four-factor general linear model (Material × Technique × Configuration × Time). Type III sums of squares were used. Model assumptions were checked (homogeneity of variance via the Levene test). Post hoc comparisons between the baseline and day 7 measurements were performed within each factor level and adjusted using the Holm procedure. For descriptive presentation of effects, model-adjusted means (emmeans) with corresponding SD values were tabulated together with letters to display groupings (α = 0.05). All analyses were conducted in R (version 4.5.1) using the “car”, “emmeans”, and “effect-size” packages. The null hypotheses of the study were that different knot configurations would not affect the maximum load to failure and elongation at failure of each type of suture material and that the knot configuration and suturing technique would not influence the mechanical properties of the sutures.

## 3. Results

### 3.1. Effect of Suturing Technique

As shown in Table 2 and Table 3, the suture technique exerted a statistically significant main effect on both failure load and elongation (*p* < 0.001). In the Material × Technique × Time interaction, the Laurell–Gottlow technique consistently produced higher failure load values than the horizontal mattress technique using nylon and polypropylene at both baseline and after 7 days of immersion (each *p* < 0.001). For PTFE, although the overall direction again favored the Laurell–Gottlow technique, this difference disappeared under Configuration C (*p* > 0.05), indicating that the effect of technique in PTFE is configuration-dependent (Table 4 and Supplementary File S1).

For elongation (Table 5 and Supplementary File S2) the horizontal mattress technique resulted in significantly higher values than Laurell–Gottlow for nylon and polypropylene at both time points (each *p* < 0.001). For PTFE, no significant difference between techniques was detected using Configurations B and C (*p* > 0.05). These results indicate that the extensibility characteristics of PTFE are less responsive to the type of suturing technique.

### 3.2. Effect of Suture Material

The material factor demonstrated a strong effect and was the predominant factor for both outcomes (*p* < 0.001; Table 2 and Table 3). PTFE consistently displayed the lowest mean failure load and elongation values at both baseline and day 7 across all techniques and configuration combinations (Table 4 and Table 5). Nylon showed the highest failure load values at baseline (Table 4), and this superiority over polypropylene was most apparent using the Laurell–Gottlow technique. In terms of elongation, nylon also demonstrated significantly higher averages than polypropylene and PTFE at baseline (*p* < 0.001 for both; Table 5), indicating that nylon initially possessed the most favorable tensile elongation profile among the evaluated materials.

### 3.3. Effect of Immersion Time

Immersion time significantly influenced both outcomes (*p* < 0.001; Table 2 and Table 3). After 7 days of immersion, nylon showed a pronounced decrease in failure load using the Laurell–Gottlow technique (*p* < 0.001; Table 4). A similar reduction was observed for nylon using the horizontal mattress technique, particularly with Configuration C (*p* < 0.001). PTFE also exhibited a marked reduction, which was most evident in Configuration B using both techniques (*p* < 0.001 for both; Table 4).

In contrast, polypropylene behaved differently under the different conditions: using the horizontal mattress technique, Configurations A and B demonstrated an increase in failure load after immersion, reaching approximately 25 N (Table 4). Regarding elongation, all three materials showed a significant reduction after immersion (*p* < 0.001 for both; Table 5). Collectively, these patterns indicate that immersion generally weakens mechanical performance, although the magnitude and direction of this effect depend on the material and configuration.

### 3.4. Effect of Knot Configuration

Configuration also showed a statistically significant effect (*p* < 0.001; Table 2 and Table 3). Configuration A tended to yield higher failure load values at day 7 across all materials and techniques. Conversely, Configuration C was associated with substantially lower failure load values after immersion, particularly when the horizontal mattress technique was applied (*p* < 0.001 for all materials; Table 4). Nylon sutures in Configuration C exhibited a significant drop in failure load values from baseline to day 7 using both techniques (*p* < 0.001 for both).

With respect to elongation, the influence of configuration for PTFE was minimal; no significant differences between configurations were observed at baseline or day 7 (*p* > 0.05; Table 5), suggesting that the elongation performance of PTFE is governed predominantly by intrinsic material characteristics rather than knot configuration.

## 4. Discussion

To our knowledge, this is the first study to investigate the effect of different knot configurations and immersion time on nonabsorbable monofilament suture materials tied using either the Laurell–Gottlow or the horizontal mattress suturing technique. We hypothesized that knot security could be improved by using different knot configurations combined with particular suturing techniques. To test this hypothesis, the two techniques were used to form three different configurations, after which the failure load and elongation values of the suture materials were measured at baseline and at the end of an immersion period. The utilization of two different suturing techniques with three knot configurations enabled an extensive comparison of the horizontal mattress and Laurell–Gottlow suturing techniques, mimicking probable real-life situations where a decision has to be made regarding the most appropriate suturing method. The major findings of this study indicate that the mechanical properties achieved by the Laurell–Gottlow suturing technique are more favorable than those achieved by the horizontal mattress suturing technique, regardless of the configuration or the suture material used.

Recent studies have advocated for the clinical use of nonabsorbable monofilament suture materials in oral and maxillofacial and periodontal plastic surgery procedures, citing the superior mechanical properties and favorable outcomes of such materials [27,28,29]. The nonabsorbable features of these materials enable them to maintain their tensile strength for longer durations instead of dissolving easily over time, as their absorbable counterparts do; thus, the use of nonabsorbable sutures in surgical fields can facilitate better healing and tissue regeneration [30]. Additionally, compared to the structures of multifilament suture materials, monofilament suture materials pose lower risks of infection due to capillary penetration by microorganisms and other foreign material [31]. However, it is more difficult to manipulate monofilament suture materials due to their greater bending stiffness while passing them through tissues compared to multifilament materials [32,33,34]. Im et al. emphasized that the lower friction coefficient observed with synthetic monofilament suture materials gives rise to concerns regarding knot security compared to multifilament sutures [35]. They also noted a scarcity of studies investigating the effects of tying methods and conditions, as well as difficulties in reproducing the outcomes and a lack of clarity regarding differences in tying techniques. Lawrence et al. similarly noted that, over the last three decades, biomechanical studies related to suture materials have focused on improving suture strength through various suturing techniques rather than on enhancing their material properties [36]. Considering the challenges related to the handling and knot security of monofilament suture materials, investigations based on different configurations and techniques could provide crucial information on knot security.

Wong and McGrouther’s comprehensive systematic review of surgical knot biomechanics analyzed 36 studies covering 24 different knot configurations. Their synthesis provides critical context for our configuration-dependent findings [8]. Specifically, they identified knot configuration as one of four primary determinants of slippage resistance (alongside suture material, size, and number of throws), with surgeons’ knot patterns consistently demonstrating superior security compared to simple square knots. Several studies have observed that the failure load of suture materials can be enhanced by using different combinations of knots and numbers of throws [18,37,38]. For example, it was reported that there was no significant difference in knot security with more than four throws, while knots with five throws had a higher tensile failure load [39]. Muffly et al. concluded that five throws could generate the failure load necessary to protect against untying [21]. Moreover, they also found that a surgeon’s knot (2 = 1) plus three additional square knots maintained the appropriate knot security for suture materials with high memory, particularly polypropylene [21]. Given that five-throw knots have been shown to provide knot security, we added three square knots using the regular direction for throws (one forward, one reverse, and one forward again) after tying each selected knot configuration: a surgeon’s knot (2 = 1), a surgeon’s knot granny (2 × 1), and a reverse surgeon’s knot granny (1 × 2).

Discussions in the literature on the failure load of suture materials used with different knot configurations, particularly in regard to whether or not the granny knot has less strength than the surgeon’s knot, have been inconclusive [17,32,35,40]. However, manufacturers provide some specific recommendations related to configurations depending on the number of throws, such as the single-throw forward, double-throw forward, and single-throw reverse configurations (1 × 2 = 1) for nylon (Resolon, Resorba Medical GmbH, Nürnberg, Germany) or the double-throw forward, single-throw forward, and single-throw reverse configurations (2 × 1 = 1) for PTFE (Cytoplast, Osteogenics Biomaterials, Lubbock, TX, USA). These recommendations can guide clinicians in the handling of specific materials while also guaranteeing knot security.

A recent study reported that although there was no significant difference in failure load between the surgeon’s knot granny (2 × 1 = 1) and the surgeon’s knot (2 = 1 = 1), granny knots are less preferred due to the common belief that they have a lower strength [40]. It was also previously indicated that the surgeon’s knot is not superior to the granny knot in terms of the properties of PTFE and polypropylene sutures [41]. On the contrary, Faulkner et al. reported that one granny knot was appropriate as the preliminary knot configurations before achieving knot security for polypropylene sutures [20]. Abellan et al. showed that PTFE sutures in a configuration with three throws forward, one forward, and one reverse (3 × 1 = 1) had higher resistance to tension than configurations with two throws forward, one reverse, and one forward (2 = 1 = 1) or one throw forward, two forward, and one reverse (1 × 2 = 1) [18]. In the same study, polyamide sutures were found to be the most resistant in a configuration with one throw forward, two forward, and one reverse (1 × 2 = 1) [18]. Based on their investigation of the features of PTFE suture materials, Hertweck et al. concluded that the proper tensile strength was achieved with basic knot configurations such as 2 = 1 = 1, whereas it was not achieved with more complex knot configurations [38]. These authors showed that such a relatively simple knot configuration could maintain a high tensile strength, making it suitable for clinical practice. Alves de Oliveira et al.’s systematic review demonstrated that knot configuration significantly influences both tensile strength and knot security independently of material type [42]. Their review of 10 studies revealed that surgeon’s knot configurations (2 = 1 = 1) provided superior tensile strength compared to alternative configurations. This result is consistent with the findings of the present study, as we demonstrated, based on a thorough examination of the characteristic features of different suture materials following immersion in artificial saliva, that the same 2 = 1 = 1 pattern (Configuration A) tended to yield higher failure load values at day 7 across all materials and techniques. Moreover, we found that the more complex throw pattern of 1 × 2 = 1 (Configuration C) was associated with substantially lower failure load values after immersion, particularly when the horizontal mattress technique was applied. PTFE was the only material that exhibited minimal change in elongation across all configurations.

This study also found that nylon had a superior failure load compared to the other suture materials at baseline for each configuration, and this superiority over polypropylene was most apparent under the Laurell–Gottlow technique. Some studies [39,43] demonstrated that the failure load of polypropylene is higher than that of nylon, while many others [44,45,46] concluded the opposite. In this study, we rejected our hypothesis that specific knot configurations are more advantageous when using nonabsorbable monofilament suture materials. For instance, nylon lost its superiority at day 7 only when complex knot configurations such as configuration C were used. Moreover, nylon’s superior performance can be attributed to strong intermolecular hydrogen bonding between its polyamide chains, which provides exceptional resistance to tensile stress and enables efficient load distribution across the knot structure [47]. The time-dependent decline in nylon’s performance reflects its hydrophilic nature; water absorption leads to plasticization of the polymer matrix, reducing its stiffness and load-bearing capacity—a well-documented phenomenon in the literature [47,48,49]. In contrast, PTFE’s low initial strength stems from its low failure load and characteristically smooth, low-friction surface, which compromises knot security. However, PTFE’s chemical inertness and hydrophobic properties confer remarkable stability over time (*p* = 0.162), making it suitable for applications where long-term biocompatibility is more important than initial mechanical strength. Additionally, polypropylene occupies an intermediate position, balancing moderate tensile strength with hydrophobic stability, though some stress relaxation was observed after immersion (*p* < 0.001). These findings underscore how polymer chemistry fundamentally governs suture performance, with material selection dependent on whether immediate strength or sustained stability is prioritized [47,48,49]. Our findings regarding time-dependent mechanical property changes align with those of Ferguson et al., who demonstrated that environmental factors significantly influence suture degradation rates in the oral cavity [50]. Moreover, the progressive mechanical property degradation observed from baseline to day 7 in our study aligns with previous studies’ findings [42,50], which documented significant tensile strength loss in sutures exposed to oral environments over time. This highlights the importance of considering environmental exposure duration when evaluating suture performance in oral maxillofacial and periodontal applications.

Several of the in vitro studies discussed above focused on issues related to both knot configurations and the mechanical properties of suture materials commonly used in surgical fields. However, there are relatively few studies comparing the effects of suturing techniques on wound healing [2,51]. The choice of suturing techniques is left to the discretion of clinicians and is often based on their dexterity in handling specific suture materials, as well as the requirements of the operation type, the specific wound, and the location [2,29,51]. For example, for guided bone regeneration procedures, it is preferable to use a deep horizontal mattress suture to release tension combined with simple-loop interrupted sutures in the superficial layer to avoid wound dehiscence [2,20,29,51]. In the present study, the Laurell–Gottlow suturing technique enabled both nylon and polypropylene suture materials to show resistance to significantly higher tension and exhibit less elongation than the horizontal mattress suturing technique. The Laurell–Gottlow suturing technique may also be preferred over the combination of the horizontal mattress and simple-loop interrupted suturing technique to save time, as the wound is crossed twice for every knot that is tied. Overall, in this study, the failure load values of the suture materials tied using the Laurell–Gottlow technique were higher than those tied using the horizontal mattress technique. Nevertheless, it is crucial to consider how PTFE suture materials maintain their failure load when tied using both techniques, particularly in the case of Configuration C.

In the present study, the tail length was rigorously controlled following established protocols. As recommended by the previous studies [15,21,22,23], all suture tails were cut to 3 mm long after knot tying. This standardized tail length was verified before each test using a ruler. This approach is consistent with the best practices in suture biomechanical testing and minimizes one potential source of variability in knot performance.

The main limitation of this study is that although the results of our in vitro research support the use of nonabsorbable monofilament suture materials with a basic configuration, such as a surgeon’s knot together with square knots, there is limited literature to confirm this configuration’s suitability. More research is needed before firm conclusions can be drawn. Specifically, this study did not include ultrastructural analysis such as scanning electron microscopy (SEM). SEM imaging would have provided valuable insights into surface morphological changes, fiber degradation, and structural alterations following immersion, potentially elucidating the mechanistic basis for the observed time-dependent changes in mechanical properties. Future investigations incorporating both mechanical testing and ultrastructural characterization would strengthen our understanding of the material behavior. Another limitation of this study is that the research was conducted in an in vitro setting, making it difficult to consider in vivo variables such as the type of saliva, the occurrence of postoperative edema, or individual hygiene habits. Using different immersion media, such as phosphate-buffered saline or simulated body fluids, may be worthwhile in the future to determine whether the effects on the suture materials are medium-dependent. Another limitation is that the knot-tying tension was controlled manually without instrumental measurement. However, manual knot tying remains the predominant technique in clinical practice, and the effect of instrumentation on knot security has been shown to be insignificant for the suture types commonly used in oral surgery [24]. While all knots were tied by a single experienced operator following a standardized protocol, the absence of quantitative tension measurements introduces potential variability in the throw tightness. This may affect the absolute values of the failure load, although comparative conclusions between suture materials and knot configurations remain valid, as all specimens were subjected to the same tying protocol. In summary, our in vitro findings should not be directly extrapolated to clinical practice without prospective validation under standardized in vivo conditions and with multiple operators.

## 5. Conclusions

These findings suggest that, under controlled laboratory conditions, the Laurell–Gottlow technique may offer mechanical advantages. However, its clinical superiority requires validation in surgical settings with standardized protocols. Among the knot configurations considered in this study, Configuration A, entailing a surgeon’s knot and three square knots, appeared to be the best choice for all the tested materials and techniques. These findings can help clinicians choose the most appropriate technique and configuration when using nonabsorbable monofilament suture materials. While this in vitro study has presented valuable information about the physical properties of the considered materials under specific conditions, future studies incorporating surface analysis (SEM) and microbiological testing would provide a more comprehensive understanding. Additionally, further clinical studies are needed to prove the advantages of specific configurations or techniques when using particular suture materials and to provide a more thorough understanding of the outcomes of using various suturing techniques.

## Figures and Tables

**Table 1:** Nonabsorbable monofilament suture materials and their properties.

Suture Material Type and Size	Brand Name	Manufacturer (Lot Number; Expiration Dates)	Suggested Throw Sequence by Manufacturer
Polypropylene with USP 4-0 caliber	Propilen^®^	Dogsan Inc., Trabzon, Turkey. (060719; February 2026)	undefined
Polyamide 6-6.6 (Nylon) with USP 4-0 caliber	Resolon^TM^	Resorba Medical GmbH, Nürnberg, Germany. (R00052331; 18 November 2025)	1. single throw forward 2. double throw forward 3. single throw reverse
Polytetrafluoroethylene (PTFE) with USP 4-0 caliber	Cytoplast^TM^	Osteogenics, Biomaterials, Lubbock, TX, USA. (RC16670A; June 2025)	1. double throw forward 2. single throw forward 3. single throw reverse

**Table 2:** Four-way ANOVA (Type III) on failure load (*n* = 10).

Effect	df	F	*p*
(Intercept)	1	84,559.29	<0.001
Material	2	6948.05	<0.001
Technique	1	6445.26	<0.001
Configuration	2	104.76	<0.001
Time	1	71.86	<0.001
Material × Technique	2	642.32	<0.001
Material × Configuration	4	38.67	<0.001
Technique × Configuration	2	14.17	<0.001
Material × Time	2	137.28	<0.001
Technique × Time	1	45.67	<0.001
Configuration × Time	2	39.79	<0.001
Material × Technique × Configuration	4	20.62	<0.001
Material × Technique × Time	2	5.61	0.004
Material × Configuration × Time	4	14.81	<0.001
Technique × Configuration × Time	2	6.36	0.002
Material × Technique × Configuration × Time	4	1.32	0.263
Residuals	324		

Type III sums of squares from a unified 4-factor model (Material × Technique × Configuration × Time).

**Table 3:** Four-way ANOVA (Type III) on elongation (*n* = 10).

Effect	df	F	*p*
(Intercept)	1	36,381.42	<0.001
Material	2	3627.83	<0.001
Technique	1	1194.99	<0.001
Configuration	2	8.09	<0.001
Time	1	83.17	<0.001
Material × Technique	2	350.77	<0.001
Material × Configuration	4	18.30	<0.001
Technique × Configuration	2	10.97	<0.001
Material × Time	2	22.66	<0.001
Technique × Time	1	23.88	<0.001
Configuration × Time	2	2.76	0.065
Material × Technique × Configuration	4	4.33	0.002
Material × Technique × Time	2	22.64	<0.001
Material × Configuration × Time	4	13.26	<0.001
Technique × Configuration × Time	2	1.10	0.333
Material × Technique × Configuration × Time	4	15.94	<0.001
Residuals	324		

Type III sums of squares from the unified 4-factor model.

**Table 4:** Simple effects on failure load (Mean ± SD) (*n* = 10).

		Baseline	Day 7	
Factor	Level	Mean ± SD	Mean ± SD	*p* (Baseline vs. Day 7)
Material × Time				
	Propilen	32.31 ± 17.64 ^b^	30.02 ± 17.61 ^b^	<0.001
	PTFE	11.77 ± 6.44 ^a^	10.69 ± 6.29 ^a^	0.162
	Resolon	37.54 ± 17.86 ^c^	35.38 ± 18.08 ^c^	<0.001
Technique × Time				
	HM	33.77 ± 17.06 ^a^	31.72 ± 17.18 ^a^	0.225
	LG	21.82 ± 15.18 ^b^	19.83 ± 14.98 ^b^	<0.001
Configuration × Time				
	A	30.71 ± 17.65 ^b^	28.67 ± 17.47 ^b^	0.018
	B	28.09 ± 17.46 ^a^	26.12 ± 17.44 ^a^	<0.001
	C	27.31 ± 16.43 ^a^	24.44 ± 13.82 ^a^	<0.001

Model-adjusted means (emmeans) with SD calculated from the corresponding raw subsets. Pairwise comparisons of baseline vs. day 7 are Holm-adjusted. Lowercase letters indicate groupings that are not statistically different within each time point (α = 0.05). Elongation values are shown without decimals for ease of interpretation. HM, horizontal mattress suturing technique; LG, Laurell–Gottlow suturing technique.

**Table 5:** Simple effects on elongation (*n* = 10).

		Baseline	Day 7	
Factor	Level	Mean ± SD	Mean ± SD	*p* (Baseline vs. Day 7)
Material × Time				
	Propilen	10,049 ± 3839 ^b^	9615 ± 3902 ^b^	0.001
	PTFE	7990 ± 3782 ^a^	7660 ± 3801 ^a^	<0.001
	Resolon	12,105 ± 3404 ^c^	11,564 ± 3527 ^c^	0.071
Technique × Time				
	HM	11,493 ± 3566 ^b^	11,050 ± 3624 ^b^	0.003
	LG	8800 ± 3415 ^a^	8333 ± 3413 ^a^	<0.001
Configuration × Time				
	A	10,424 ± 3844 ^a^	10,039 ± 3921 ^b^	<0.001
	B	9570 ± 3631 ^a^	9131 ± 3625 ^a^	<0.001
	C	4512 ± 2317 ^a^	4160 ± 2363 ^ab^	<0.001

Model-adjusted means (emmeans) with SD calculated from the corresponding raw subsets. Pairwise comparisons of baseline vs. day 7 are Holm-adjusted. Lowercase letters indicate groupings that are not statistically different within each time point (α = 0.05). Elongation values are shown without decimals for ease of interpretation. HM, horizontal mattress suturing technique; LG, Laurell–Gottlow suturing technique.

## Data Availability

The original contributions presented in this study are included in the article/Supplementary Materials. Further inquiries can be directed to the corresponding author.

## Supplementary Materials

The following supporting information can be downloaded at https://www.mdpi.com/article/10.3390/jfb16120428/s1, Supplementary File S1: Failure_KeyContrasts; Supplementary File S2: Elongation_KeyContrasts.

## Abbreviations

The following abbreviations are used in this manuscript:

PTFE	polytetrafluoroethylene
N	newton
µm	micrometer
mm	millimeter
